# 

**Published:** 1871-07

**Authors:** 


### INDEX OF SUBJECTS.

### ORIGINAL COMMUNICATIONS.

Gynæcological Medicine........................................................337

Treatment of Croup.........................................................348

Report of five cases of Typhus Fever treated with Belladonna.................350

The use of Raw Meat..............................................................352

Treatment of Sciatica..........................................................353

Catarrhal Pneumonia in Infants at the Breast...............................354

### EXTRACTS AND GLEANINGS.

Epilepsy—Hypodermic Syringe—Local application of Ice and Cold Water in Scarlatina, Diptheria, Croup, etc. 355; New uses of Arnica Montana—Turpentine in Pupura Hemorrhagia, 356; On the treatment of Scrofulous Diseases—Treatment of Chorea—Treatment of Leucorrhœa—Vomiting of Pregnant Women—Croup, 357; Coffee as a powerful Antidote—Radical cure of Hydrocele—New mode of inducing vomiting—Chloroform Ointment—Gangrenous Ulcers—Senile Gangrene, 358; Citrate of Caflein in Neuralgia—Chionanthus Virginica—Bromide of Potassium and Ammonium in Insomnia—Chloride of Zinc Paste, 359; On the use of Arsenic in certain painful affections of the Stomach and bowels; Bromide of Potassium in Hydrophobia, 360; Lactate of Iron—Urethral Stricture—Calabar Bean in Suppuration of the Cornea—Galactorrhœa, 361; Carbolic Acid in Phthisis—Digitalis in Pneumonia—Anti-Spasmodic Mixture—Potion for Diarrhœa—Belladonna as a local application—Carbolic acid as an Inhalation—Nervous Aphonia—Prurtus Pudendi—Liniment for Sprains, etc., 362; Iodine—Iodine in Incontinence of Urine in old people Comp. Ch. Pectoral, 363; Sleeplessness during Fever—The use of Camphor in Hospital Gangrene—Ioduret of Silver in Whooping Cough—On the treatment of Venereal Vegetation, 364; the use of bath in fever—for Dysmenorrhoea, 365; Whooping-Cough—To remove stains of Silver—Dr. Hebeden's treatment of Dysentery—Internal use of Nitrate of Silver—Muriate Tincture of Iron, in Erysipelas—Exanthemic Liniment—Digitalis in Earache, 366; M. Velpeaus' new caustic—Astringent or anti Diarrhoea Medicine—Chloral Hydrate in Nocturnal Enuresis—Excessive Typanitic Distension, 367; Iodide of Potassium in Bright's Disease—Bromide of Potassium in Uterine Fibrids—Styptic Colloid, 368; Ulcers a cause of Bright's Disease—New method of reducing dislocations of the Shoulder—The Influence of Abnormal Milk on Nutrition—Sulphate of Iron in Suppuration—Purification of Hydrate Chloral, 369; Chloral in Midwifery, 370; Antiphlogistic value of Ergot—Management of Scabies, 371; Puncture of the Abdomen for the relief of Tympanitis—Chloral in Delirium Tremens—The local and superficial anæsthetic effects of the Phenols 372; on the removal of subcutaneous tumors without hemorrhage or loss of skin—Aconite is the child's remedy—Shampoo for Baldness—Toothache, 373.

### CORRESPONDENCE—HYGENIC AND MISCELLANEOUS.

Proceedings of the Medical Convention at Macon..............................374
Fetid Breath......................................................................381

### EDITORIALS, REVIEWS, ETC.

Our Journal.......................................................................382
The Atlanta Medical College and its Apologists..............................384
Proceedings of the Medical Convention.........................................388
A Pleasant Time..................................................................390
Our College Friends.............................................................391
A Flattering Testimonial—A word to our Subscribers.......................392
Editorial Items..................................................................393
The College Controversy—Books and Pamphlets.............................394

